# Factors influencing growth hormone levels of Bali cattle in Bali, Nusa Penida, and Sumbawa Islands, Indonesia

**DOI:** 10.14202/vetworld.2017.1250-1254

**Published:** 2017-10-18

**Authors:** N. K. Suwiti, I N. K. Besung, G. N. Mahardika

**Affiliations:** 1 Bali Cattle Research Center, Udayana University, Jl. Sudirman, Denpasar 80226, Bali, Indonesia; 2 Department of Bacteriology, Faculty of Veterinary Medicine, Udayana University, Jl. Sudirman, Denpasar 80226, Bali, Indonesia; 3 Department of Animal Biomedical and Molecular Biology, Faculty of Veterinary Medicine, Udayana University, Jl. Sudirman, Denpasar 80226, Bali, Indonesia

**Keywords:** Bali cattle, growth hormone, Indonesia

## Abstract

**Aim:**

Bali cattle (*Bos javanicus*) are an Indonesian’s native cattle breed that distributed in Asia to Australia. The scientific literature on these cattle is scarce. The growth hormone (GH) of Bali cattle is investigated from three separated islands, namely, Bali, Nusa Penida, and Sumbawa.

**Materials and Methods:**

Forty plasma samples were collected from each island, and the GH was measured using a commercial enzyme-linked immunosorbent assay kit. The data were analyzed based on the origin, sex, and cattle raising practices.

**Results:**

We found that the GH level (bovine GH [BGH]) of animal kept in stall 1.72±0.70 µg/ml was higher than free-grazing animal 1.27±0.81 µg/ml. The GH level was lower in female (1.22±0.62 µg/ml) compared to male animals (1.77±0.83 µg/ml).

**Conclusion:**

We conclude that the level of BGH in Bali cattle was low and statistically equal from all origins. The different level was related to sex and management practices. Further validation is needed through observing the growth rate following BGH administration and discovering the inbreeding coefficient of the animal in Indonesia.

## Introduction

Bali cattle (*Bos javanicus*) [1,2], an indigenous cattle in Indonesia and a domesticated descendant of the wild banteng (*B. javanicus*), represents about 27% of the total cattle population in Indonesia [3,4]. The cattle are also found in southern Asia, Southeast Asia, up to Australia, and even Hawaii [5]. Bali cattle are one of the important beef cattle breeds contributing to the development of livestock industries in Indonesia and are the most predominant genotype within the eastern islands, for example, in Bali, West Nusa Tenggara, and East Nusa Tenggara provinces [6].

International publications for these unique cattle are scarce. The Bali cattle’s raising varies in Indonesia. It expands from intensive (stall) to extensive (pasture) and mixed intensive and extensive. The latest means that the animal is kept partially in a stall with irregular free grazing. The growth performance of Bali cattle is considered to be low. Their body weight grows only around 0.3 kg/day [7]. The body weight increases to a maximum of around 300 kg at the puberty than stagnant thereafter [8]. In comparison, another cross Brahman and Simmental breeds grow around 1.3-15 kg BW/day [9]. Performances of a breed or crossbred cattle’s are not expected to be the same under all environments [10]. The significant differences found between the Bali cattle from Bali island and the ones from Lombok island were all related to the body measurement, and it indicates that the differences in size might not only be due to management system but also to genetic factors. However, the much smaller size of the Lombok bulls is most likely a result of both management and genetic status [11].

We hypothesized that the discrepancy in growth performance is due to different level of bovine growth hormone (BGH). BGH plays a very important role in many physiological actions [12]. Growth hormone (GH) has wide physiological activities, which include the regulation of growth, lactation and mammary gland development, gluconeogenesis, the activation of lipolysis, and the enhancement of amino acid incorporation into muscle protein [13]. GH directly or indirectly plays a notable role in tissue growth and fat metabolism. Thus, it has an important role in reproduction, lactation, and growth stimulation in animals [14,15]. Here, we describe the level of BGH in Bali cattle in various sexes, method of raising, and origin in Bali, Nusa Penida, and Sumbawa islands. The three separated islands represent different land fertility and cattle raising practices. In Bali, the animal is kept install, in Nusa Penida, it is mixed stall and free grazing, while in Sumbawa, it is free grazing what locally known as “*lar*.”

## Materials and Methods

### Ethical approval

The ethical clearance for this study is evaluated and provided by the Faculty of Veterinary Medicine, Udayana University.

### Location

The samples were from Bali, Nusa Penida, and Sumbawa islands.

### Samples

Eighty plasma samples of Bali cattle’s from each area were selected using random sampling in five villages in Nusa Penida, namely, Tanglad, Bunga Mekar, Sauna, Batu Madeg, and Ped, five villages in Sumbawa, namely, Moyo Utara, Moyo Hilir, Unter, Iwes, and Moyo Hulu, and five villages in Bali, namely, Sudimara, Catur, Kaliasem, Rendang, and Kusamba. The whole blood was drawn from a jugular vein and collected using Venoject 10 ml vacuum tube with disodium ethylenediaminetetraacetic acid as an anticoagulant.

### BGH measurement

The BGH was measured using BGH kit Cloud-Clone Corp (Buckingham, UK) which was previously applied [14]. The supplied protocol was strictly applied. Briefly, 50 μl of each sample was mixed with the same volume biotin labeled GH and incubated for 1 h in 37^°^C. To prepare the standard curve, the standard unlabeled GH was serially diluted in PBST ranging from 100 to 0.1 ng/ml. Fifty microliters of each dilution were mixed with labeled GH in duplicate. The plates were incubated for 1 h at 37^°^C after which these were washed thrice with PBST as before. The horseradish-peroxidase labeled streptavidin provided in the kit (reagent B) in PBST was then loaded to the wells and incubated for 1 h at 37^°^C. Finally, the wells were washed 5 times with PBST, and 90 μl/well of the substrate provided in the kit was added. The plates were then incubated finally for 10 min at 37^o^C, and the enzyme reaction was stopped by adding provided stop solution. The absorbance was recorded in an enzyme-linked immunosorbent assay reader Multiskan Thermo Scientific at 450 nm against a blank with no PBST.

## Results

**Figure-1:**
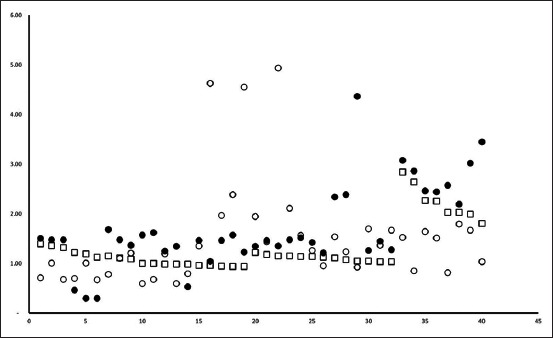
The scattered plot of bovine growth hormone level of serum of Bali cattle from Bali (filled circle), Sumbawa (unfilled circle), and Nusa Penida (unfilled box).

**Figure-2:**
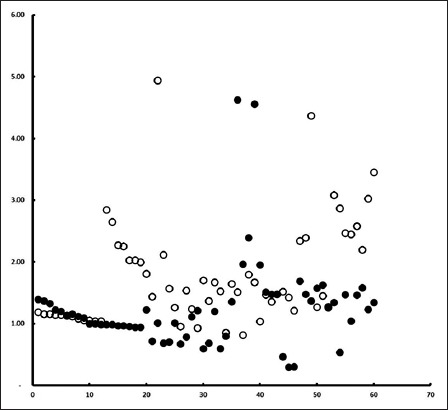
The scattered plot of bovine growth hormone level of serum of Bali cattle of female (filled circle) and male (unfilled circle) animals.

**Figure-3:**
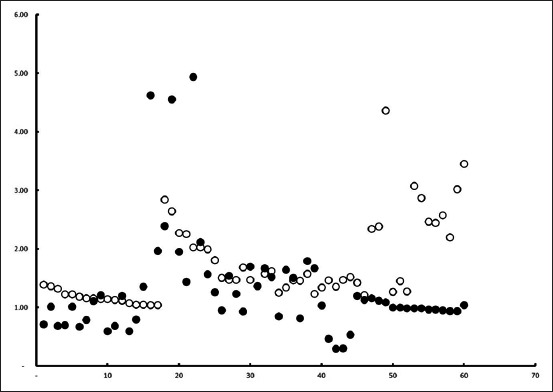
The scattered plot of bovine growth hormone level of serum of Bali cattle of free grazing (filled circle) and install (unfilled circle) animal raising.

Overall, BGH plasma concentration was 1.50±0.78 µg/ml. Average concentration of BGH in the plasma of Bali cattle of different origins, sex, and cattle raising is presented in Table-1. Table-1 summarizes that the level of BGH is independent of the origin (p=0.125) and dependent on sex (p=0.000) and cattle raising practice (p=0.007). In other words, we detected no statistical difference of BGH level in various origin, while the level was statistically higher in male than female (p=0.000), as well as higher in cattle raised install than free grazing (p=0.007). The scattered plot graphic of individual cattle based on origin, sex, and cattle raising method is presented in Figures-1-3, respectively. The figures indicate that the individual BGH level was mixed which tends to be higher in male cattle (Figure-2) and stall cattle raising system (Figure-3).

**Table-1:** Average concentration of bovine growth hormone in the plasma of Bali cattle of different origins, sex, and cattle raising.

Parameter	Description	Number of samples	Average	p
Origin	Bali Island	40	1.33±0.49	0.125
	Nusa Penida Island	40	1.50±0.10	
	Sumbawa Island	40	1.70±0.84	
Sex	Female	61	1.25±0.74	0.000
	Male	59	1.77±0.83	
Cattle raising	In stall	61	1.72±0.69	0.007
	Free grazing	59	1.30±0.90	

## Discussion

Research and international scientific publication on many aspects of Bali cattle need to be enhanced to provide a strong database for its development and its availability for international audiences. This breed is predominant in Indonesia, due to its resilience in tropic condition [16]. It is a direct descendant of wild banteng (*B. javanicus*) into Bali cattle [1,2] which is still available in Indonesia, especially in Baluran National Park in East Java. This manuscript is the first scientific publication on BGH level of this breed. We found that the level of BGH is generally lower than any reference data. The average of plasma BGH was 1.50±0.78 µg/ml. Published ranges of GH of various cattle are 1.7-3.3 µg/ml [14,17]. This fact is presumable the basis of low growth rate of Bali cattle. An experiment to justify this hypothesis needs to be conducted to compare the growth performance of BGH-treated animal with untreated one.

Bali cattle are endemic animal in various islands in Indonesia, especially Bali. This island is thought to be the origin of Bali cattle domestication and provide a source of cattle to be distributed to other islands in Indonesia, and hence, its common name originated [16]. The population in Bali is kept intact, as there is a regulation that it is not allowed to import Bali cattle to Bali. This fact might lead to lowering the genetic variability of Bali cattle which generate a population with high inbreeding event [2]. Research on the genetic structure of Bali cattle in Bali needs to be conducted to reduce the chance of genetic inbreeding [18]. Inbreeding is mostly causing the reduction of genetic quality of animal [19,20].

The homogeneity of BGH level in three separated islands under the study shows the possible common origin of Bali cattle in those islands. We hypotheses, the discrepancy of soil fertility and composition should lead to different BGH level. We provide evidence that it is not the case. The BGH level of cattle from Bali, Nusa Penida, and Sumbawa islands is statistically homogenous (Table-1 and Figure-1).

However, the BGH level does differ in sex and cattle raising practices. Male cattle have average BGH level of 1.77±0.83 µg/ml, while female cattle have a lower average of 1.22±0.62 µg/ml. This is a common sense in animal research. The sex factor influences the production of GH, in which male animal produces a higher level of this hormone than that of female [21]. The higher growth rate of male than female Bali cattle has previously been reported [22].

We investigated the BGH level install and free-grazing animal raising practices. We strictly avoided the mixed farming system, due to the irregularity of keeping animal in the stall and free grazing. We found that the BGH level of animal kept in stall 1.72±0.70 µg/ml was higher than free-grazing animal 1.27±0.81 µg/ml. This largely results from poor nutrition of cattle managed under traditional smallholder feeding systems which rely on communal grazing of overstocked and weedy native pastures [23]. Production of GH is modulated by many factors, including stress, exercise, and nutrition [24]. The Bali cattle in Bali Island are managed under different and better conditions compared to other areas in Indonesia. Its, therefore, have a higher weight and do not seem to have the same weaknesses such as slow growth rate, small body size, and high calf mortality as Bali cattle on other locations in Indonesia. A study conducted by Mastika [25] suggested that differences within the breed may not only be related to genetic factors but also environmental, nutritional, and management factors [26]. Bali cattle raised install are fed twice a day with *ad libitum* drinking water and additional nutrients of polar and mixed mineral milk [27]. In addition to being a major source of dietary calcium, milk may also raise the level of insulin-like growth factor-I (IGF-I) [28]. The somatotropic axis, consisting of GH, hepatic IGF-I, and assorted releasing factors, regulates growth and body composition [29]. The GH is also known as an appetite-regulating hormone [30].

The low level of serum BGH seems merely to be caused by genetic factor. Bali, Nusa Penida, and Sumbawa Islands are different in plantation, rainfall, and soil physicochemical properties [23,31].

It was plausible to draw a hypothesis that the BGH level in those islands is statistically different. The result shows that it is not the case. There is no significant difference of BGH in Bali cattle’s grown in Bali, Nusa Penida, and Sumbawa Islands. This indicates that the performance of the breed is not strongly influence of pasture quality.

One genetic factor that leads to the low performance of Bali cattle is inbreeding event. Bali cattle are endemic in Indonesia and were originated from Bali or East Java, where its origin, wild Banteng, came from. We assume that the domestication might have happened recently, as Bali cattle looks indifferent to its ancestor and it sometimes still exhibits its wild characteristic (data not shown). Endemic animal has been proofed to be inbreeding prone as proofed in wild animal [32]. This seems to be valid for domesticated animal, which is endemic in the small area. This needs to be elucidated in Bali cattle through investigating the inbreeding coefficient throughout the country.

## Conclusion

The level of BGH in Bali cattle was low and statistically equal from all origins. The different level was related to sex and management practices. Further, validation is needed through observing the growth rate following BGH administration and discovering the inbreeding coefficient of the animal in Indonesia.

## Authors’ Contributions

All authors designed this research. NKS designed the experiment. NKS and INKB collected field samples. NKS, INKB, and GNM conducted the laboratory testing. INKB and GNM prepared the data sets. NKS, INKB, and GNM drafted the manuscript. All authors read and approved the manuscript.
